# A Novel Pressure-Assisted Induction Melting Technique for Synthesis of Lightweight High-Entropy Alloys: A Concept, Process Development and Hardware Design

**DOI:** 10.3390/ma19081588

**Published:** 2026-04-15

**Authors:** Peter Newcombe, Frank Czerwinski

**Affiliations:** CanmetMATERIALS, Natural Resources Canada, Hamilton, ON L8P 0A5, Canada

**Keywords:** lightweighting, high-entropy alloys, alloy synthesis, vacuum induction melting, pressure-assisted induction melting

## Abstract

Lightweight high-entropy alloys are primarily designed to overcome the strength-to-density ratio limitations of conventional counterparts and often consist of elements with drastically different melting temperature and vapor pressure. Their chemistry, therefore, imposes challenges on alloy synthesis, particularly through liquid metal engineering routes, since elements with high vapor pressure (e.g., Mg, Zn, Li) vaporize before the higher-melting-point ingredients (e.g., Cu, V, Ni) are fully molten, resulting in volatile element loss. To overcome this challenge, a novel pressure-assisted induction melting (PAIM) process was developed and the proprietary furnace for its implementation was designed and built. The system allows precision melting of up to 10 cm^3^ of an alloy at temperatures up to 1700 °C while addressing the partial pressure requirements during the melting progress. The chamber is prepared using rough vacuum and re-filled with inert gas such as argon with the operating pressure range from about 10^−4^ MPa up to maximum of 1.6 MPa (233 psi). The alloy chemical composition can be modified in situ by feeding solid additives at specific melting stages through the isolated airlock without disrupting the pressure conditions within the chamber. The viability of the concept was verified by synthesis of two lightweight non-equimolar high-entropy alloys: Mg-rich Mg_50_(MnAlZnCu)_50_ and Al-rich Al_35_Mg_30_Si_13_Zn_10_Y_7_Ca_5_. The experiments showed that sequential multi-step melting procedures, designed based on inputs from FactSage computational analysis, when combined with PAIM synthesis, allowed manufacturing fully dense and chemically homogenous complex alloy compositions with optimal volumes for materials discovery research.

## 1. Introduction

Lightweight materials have been extensively used for decades, not only in all forms of transportation but also in general applications of civil engineering and clean energy technologies [1]. Since, for the conventional alloy matrix, the compositional possibilities of properties improvement reached their limits, there is a growing interest in high entropy compositions, where the multi-principal element concept challenges the existing strategies and drastically expands alloy developmental opportunities. Lightweight high-entropy alloys (LWHEAs), including Multi-Principal Component Alloys (MCAs) and Compositionally Complex Alloys (CCAs), are designed by combining elements in equiatomic or nearly equiatomic quantities with a primary goal of addressing the critical weight challenge and overcoming the strength-to-density ratio limitations of their conventional counterparts [2,3].

There is no strict definition of LWHEAs and generally alloys with a density below 6 g/cm^3^ or sometimes even 7 g/cm^3^ are included in this group. According to a recent classification that links LWHEAs with their potential engineering applications, they are divided based on density analogies with conventional lightweight alloys [4]. For the magnesium class with densities below 2 g/cm^3^, the principal elements include Al, Mg and Li, often with Si, Ca, Zn and Cu. Examples with the lowest density include the Al_19.9_ Li_30_Mg_35_Si_10_Ca_5_Y_0.1_ alloy (1.57 g/cm^3^) with a high specific strength of 327 KPa·m^−3^ and the Al_15_Li_35_Mg_35_Ca_10_Si_5_ (1.44 g/cm^3^) alloy which shows good compressive plasticity [5]. It should be noted that for the majority of over 130 Mg-containing LWHEAs, MCAs and CCAs identified, combined with 26 heavier elements, their density is beyond the Mg class and may reach 6.0 g/cm^3^ [6]. The aluminum class HEAs with densities between 2.0 g/cm^3^ and 3.0 g/cm^3^ contain Mg in addition to Al, along with Cu, Li, Mn, Ti, Sc, and Zn. As an example, the Al_3_Mn_0.2_Zn_0.3_Mg_0.6_Si_0.7_ alloy (2.86 g/cm^3^) shows a brittle structure with a compression strength of 486–618 MPa and hardness of 268–283 HV [7]. The titanium class HEAs, displaying densities between 3.0 g/cm^3^ and 6.0 g/cm^3^, contain Al, Mg and Ti, frequently accompanied by Cu, Cr, Fe, Li, or Zn. The Li_05_Al_20_Mg_10_Ti_30_Nb_35_ (5.0 g/cm^3^) alloy, designed through computational multicomponent optimization, is an example [8].

While the density reduction in LWHEAs is achieved through the presence of Mg, Al, Li or Ti, to increase strength generally heavier elements are added. As a result, for some alloys an over 20 times difference between densities of elemental ingredients occurs, e.g., from alkali metal Li of 0.53 g/cm^3^ to transition metal Mo of 10.28 g/cm^3^. The element density differences are accompanied by extreme differences in melting temperatures and vapor pressure, imposing challenges on their synthesis. The vast majority of LWHEAs described in the literature are synthesized at the laboratory scale in very limited quantities of the order of tens of grams and the synthesis method is most often the only information provided. To address this challenge, the new synthesis technique of LWHEAs called PAIM was developed based on extensive experience with the casting of a variety of conventional alloys at the laboratory and semi-industrial scales. In this report the PAIM process and the hardware for its implementation are described. To make the report useful to a broad audience of scientists involved in researching HEAs with no casting expertise, the fundamentals of liquid metal behavior were added.

## 2. Synthesis Routes of LWHEAs: Defining the Challenges

**Figure 1:**
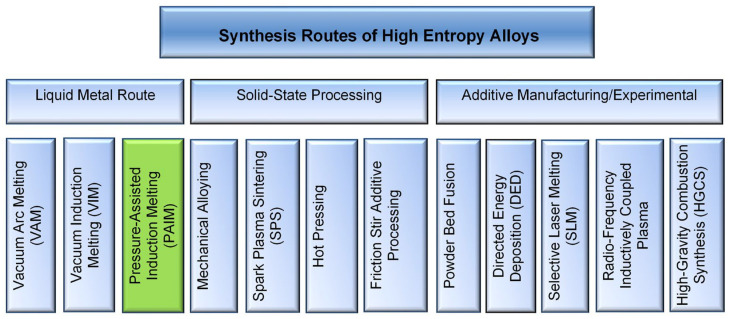
Synthesis techniques of HEAs with several experimental processes indicated. The PAIM technique developed in this study to synthesize LWHEAs is marked green.

The present routes of HEA synthesis include liquid metal routes, solid-state routes of mechanical alloying, and additive manufacturing with the applicability of individual methods being limited to specific chemical compositions [4,9]. HEAs can be generated as powders, fibers, films and bulk forms, and the latter materials are of primary interest. A selection of presently available synthesis techniques is listed in Figure 1, where the PAIM technique developed in this study is also included. In addition to conventional well-established processes, there is a search for novel synthesis methods. As an example, a new method called high-gravity combustion synthesis (HGCS) followed by post-treatment was applied for the Cr_0.9_FeNi_2.5_V_0.2_Al_0.5_ alloy, where thermite powder was used as the raw material [10]. Another experimental technique, called the radiofrequency inductively coupled plasma (RF-ICP), provides a plasma source, in which a radiofrequency current is passed through a load coil, generating an intense electromagnetic field inside the torch ionizing the gas [11]. In the search for high-throughput synthesis techniques new hardware was designed in conjunction with electric field assisted sintering (EFAS) [12,13]. Moreover, the easy synthesis routes of HEAs focus on reducing high energy consumption and bypassing complex, expensive equipment [14]. As another approach, the sustainability aspects of HEA manufacturing are considered when using different raw material sources during primary, secondary, and tertiary synthesis cycles [15].

The liquid metal routes are the most widely used because of their high processing efficiency and potential of scalability. According to the recent assessment of HEAs destined for nuclear applications, the most frequent fabrication methods were vacuum arc melting (VAM)—73%—and vacuum induction melting (VIM)—15%—with only 11% using laser-based additive manufacturing and less than 5% remaining for solid-state manufacturing [16]. In contrast to solid-state routes during liquid state synthesis, HEAs are subjected to a phase transformation as they solidify and, depending on cooling rates, they may reach either the equilibrium or non-equilibrium state.

The technique of VAM utilizes a high-energy electric arc to melt materials in a high-vacuum environment, allowing precise control over alloy composition. It minimizes contamination, is fast and works well with pure and high melting point metals [17]. The process is good for reactive metals like Ti but cannot be used with volatile metals like Zn. Depending on hardware design, there may be limitations on molten alloy stirring. In contrast, the VIM process uses electromagnetic coils for generating eddy currents in the metal charge to melt elements in a crucible environment of vacuum. In general, induction melting may be applied to relatively high liquidus temperatures and can handle elements which are reactive during melting, with residuals of the charge materials becoming important. Examples of VIM-synthesized HEAs include AlCr_1.3_TiNi_2_ with a density of 6.42 g/cm^3^ [18] and anomalous eutectic Al_21_Ti_21_V_16_Ni_21_Co_21_ with a density of 6.19 g/cm^3^ [19]. A comparison between the Ti_16.6_Zr_16.6_Hf_16.6_Co_10_Ni_20_Cu_20_ alloy synthesized by VAM and VIM concluded that VIM could achieve a similar level of homogenization in substantially less time than that observed after VAM synthesis [20].

LWHEAs have many unique features, often requiring unconventional synthesis. This is particularly true for highly efficient liquid metal routes where the key challenges are drastic differences in melting temperature and vapor pressure of elemental ingredients. As emphasized in Table 1, low-melting-point elements (e.g., Zn, Mg, Li) can vaporize before the higher-melting-point ingredients (e.g., Fe, Ni, Cu, Ti) are fully molten, leading to volatile element loss through selective vaporization. In addition, some elements may exert solubility limitations in the molten state. To achieve the targeted chemical composition and homogeneous microstructure of LWHEAs, most often designed through computer-aided methods, the liquid-state route requires a specific synthesis process and unique furnace allowing control of the melting chamber pressure.

## 3. PAIM Process Development for LWHEA Synthesis

To avoid element loss during liquid state synthesis, the molten alloy chemistry, temperature and chamber pressure should be correlated. The vapor pressure data are important to predict the loss of alloying elements due to vaporization during alloy synthesis [21]. The vapor pressure of a metal is the pressure exerted at a given temperature by the gaseous phase when a liquid metal is in equilibrium with its own vapor. The vapor pressure depends on both the element and the temperature.

**Figure 2:**
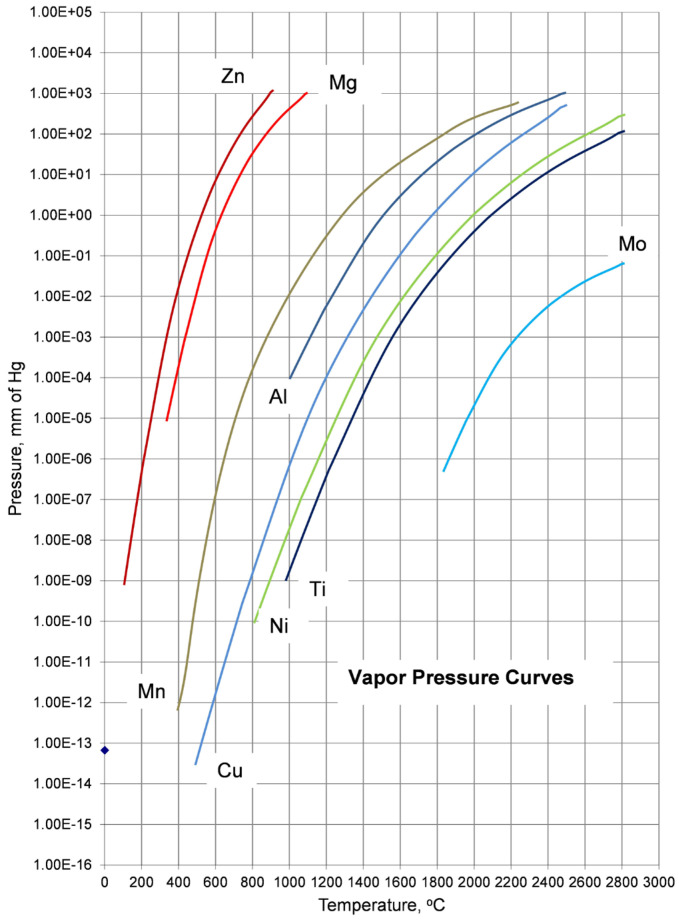
Approximate vapor pressure curves for metals commonly used in LHEAs; curves selected from reference [25].

The vapor pressure of any metal increases non-linearly with temperature, often described by the Clausius–Clapeyron relation [24]:

(1) $$\ln\frac{p1}{p2}=-\frac{∆Hvap}{R}\left(\frac{1}{T2}-\frac{1}{T1}\right)$$

where Δ*Hvap* is the enthalpy of vaporization in J/mol and is assumed to be independent of temperature, *P*1 and *P*2 are pressures in atm, at temperatures *T*1 and *T*2 in Kelvin, respectively. Examples of vapor pressure curves selected from the literature [25] for typical elemental ingredients of LWHEAs are shown in Figure 2. The area to the left of each curve represents the conditions of temperature and pressure under which the metal exists as a solid. The area to the right of each curve represents conditions where the metal exists as vapor. The major differences are seen in Mg and Zn as compared to other potential ingredients of LWHEAs.

Partial pressure is that portion of the total pressure exerted by each gas constituent within the system. During alloy synthesis, the partial pressure of the furnace atmosphere is a parameter, often set to a balance point to remove unwanted gases achieved by a high vacuum while an inert gas introduced to the chamber prevents boiling and excessive evaporation of volatile alloying elements. Partial pressures are usually introduced at temperatures well above ambient during the heat-treating cycle to ensure the removal of any residual impurities and moisture from the furnace chamber during the initial pump-down and heat-up sequence. For example, the evaporation loss of Al during melting was reduced by introducing argon gas into the furnace to increase the chamber pressure. During melting of the AlV55 master alloy, increasing the argon pressure to 2000 Pa from a vacuum of 60 Pa reduced the evaporation loss of Al from 11.48% to 0.58% [26].

As revealed above, during the synthesis design of LWHEA compositions, the melting point, boiling point and vapor pressure are the essential physicochemical data considered. For alloys containing elements with high differences between melting temperature and vapor pressure, the synthesis process requires sequential melting rather than mixing all elements at once. Sequential alloying is an advanced, step-by-step approach to create complex alloys with an objective to reduce the melting temperature of intermittent alloy components to avoid evaporation losses. To implement this concept, the alloy synthesis is divided into stages with high vapor pressure metals being added later. To reach the alloy composition target and avoid volatile element depletions, the extra amount of that element, calculated using volatility models based on physicochemical data, is added to the furnace charge. This is referred to as alloy recovery and percentages less than 100% recovery indicate that element depletion is expected. In practice, sequential melting is combined with controlling the furnace chamber pressure to prevent volatile element loss and reach the target composition of LWHEAs.

## 4. PAIM Furnace Design

**Figure 3:**
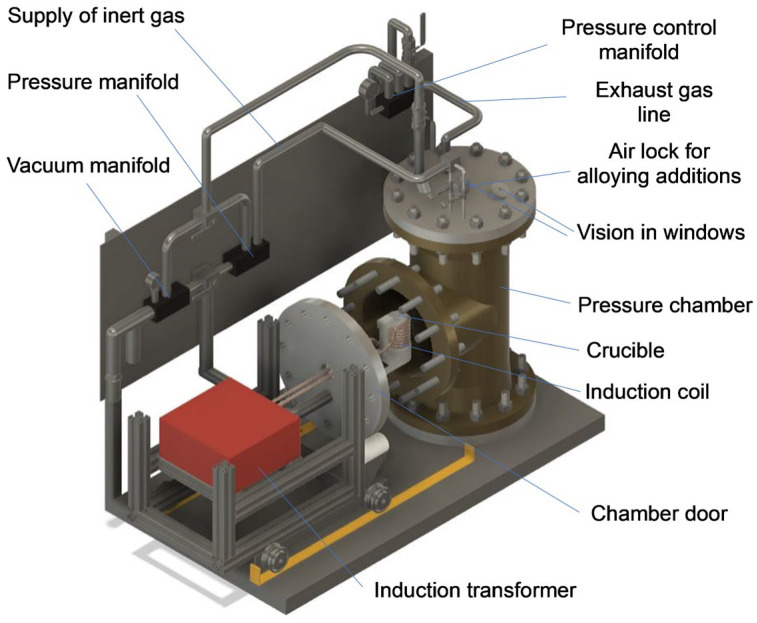
Computer-aided design (CAD) model of the PAIM furnace with indicated major components. Pressure chamber dimensions: internal diameter—254 mm; height—630 mm; volume—37 L (0.037 m^3^).

To implement the PAIM process, the proprietary furnace was designed and built. Due to advantages of induction heating, the VIM platform was selected as the base for the new hardware developed in this study. The essential part of the PAIM furnace is the stainless-steel pressure chamber with an internal diameter of 254 mm, height of 630 mm and a volume of 37 L (0.037 m^3^) (Figure 3). The side chamber door is used to insert the induction coil and ceramic crucible. The chamber was fabricated using a commercially available schedule 10 ‘T’ fitting of 304 stainless steel. Welded flanges with blind flange plates were used for end covers—all these items being pressure-rated with well-established performance. The overall maximum allowable working pressure of 1.6 MPa (233 psi) was determined by the pressure rating of the weakest component. The chamber volume was determined through our laboratory experiments as optimal for HEA development research. This volume allows for easy processing of 100–200 g of an alloy, depending on its density. The furnace induction coil is powered by a 5 kW/25kHz induction power supply, allowing fast and direct heating of the alloy charge, loaded in the crucible up to the required processing temperature. The maximum furnace temperature limit is determined by the crucible material chemistry and for the alumina (Al_2_O_3_) crucible used in the present setting it is 1700 °C, with a safety margin of 50 °C below the crucible rating of 1750 °C.

The upper flange of the pressure chamber houses ports for the exhaust gas line connected to the vacuum pump and the vacuum and argon supply enters at the side cover below the induction coil with the latter being connected to a pressurized inert gas (argon) cylinder. Both the vacuum and gas supply lines are equipped with pressure sensors. The system has capabilities to control the chamber pressure from a rough vacuum of about 10^−4^ MPa (few millibars absolute pressure) up to a maximum of 1.6 MPa pressure of inert gas. The chamber pressure is limited by two pressure relief valves for redundancy. One is set just above the desired process pressure, while the other remains fixed at the maximum allowable working pressure of 1.6 MPa to protect the chamber. The airlock also installed on the upper flange provides access for feeding alloying elements that could be inserted at certain stages of the synthesis. Alloying additions can be provided repeatedly through the airlock without disrupting the pressure conditions within the chamber and without introducing oxygen. Two viewing windows allow direct observation of the melting progress.

**Figure 4:**
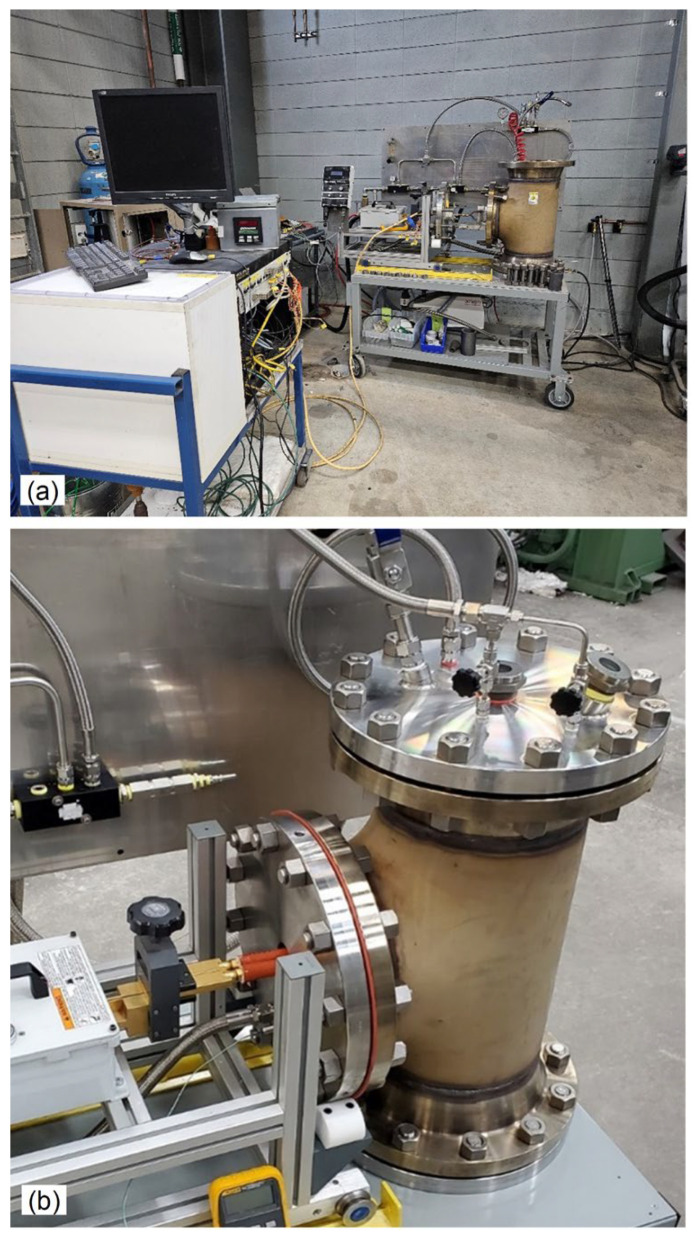
PAIM furnace built for synthesis of LWHEAs: (**a**) general view of the synthesis cell with auxiliary control equipment and gas supply; (**b**) furnace chamber with major components, indicated on the CAD model in Figure 3.

**Figure 5:**
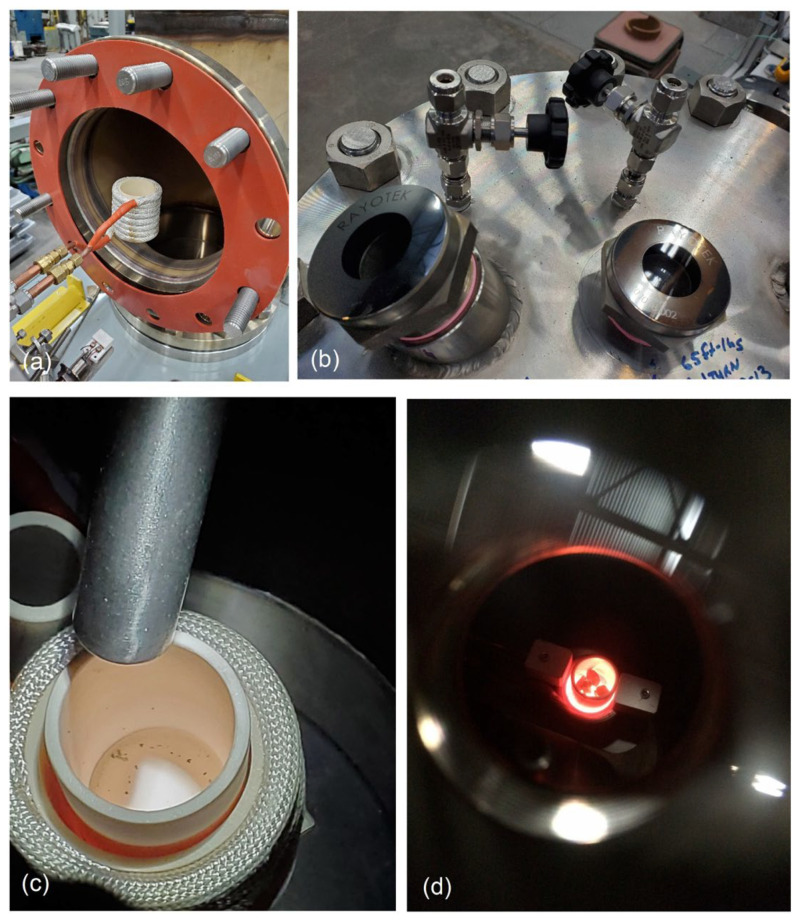
Details of the PAIM furnace design: (**a**) induction coil at the entrance to the pressure chamber; (**b**) pressure chamber top flange with air lock and two vision windows; (**c**) design of dosing additions to molten alloy through the isolated airlock; (**d**) molten alloy in the crucible as observed through the vision window.

The laboratory cell, assembled to incorporate the PAIM furnace with auxiliary equipment including pressure and temperature computer control and monitoring, water cooling system, and argon gas supply, is shown in Figure 4a. The PAIM furnace built in-house is shown in Figure 4b. Due to the size of the furnace pressure chamber, there are opportunities for future expansion of the synthesized alloy volume to accommodate larger samples up to 100 cm^3^. When changing the transformer, induction coil and crucible size, the synthesized alloy volume may be scaled up to the specific requirement. As shown in Figure 5a, the pressure chamber door size allows for the accommodation of a larger induction coil and crucible. Design details allowing feeding additions to the molten alloy and visual assessment of the crucible are shown in Figure 5b,c with an image of molten alloy observed through the vision window depicted in Figure 5d.

Since the LWHEA microstructure can be modified using the solidification rate of the alloy, the system should have the capability of changing the crucible cooling rate. In its present design, the alloy cooling rate can be increased by higher water flow through the induction coil when the power is shut down. To slow down the alloy cooling rate, a continuing very low induction heating input of approximately 0.5 kW can be used.

## 5. Verification of the PAIM Synthesis Concept

To test the PAIM furnace capabilities, two lightweight high-entropy alloys of various densities and non-equimolar compositions, Mg_50_(MnAlZnCu)_50_ and Al_35_Mg_30_Si_13_Zn_10_Y_7_Ca_5_, were selected. Both alloys contain Zn and Mg which have a very high vapor pressure in combination with a variety of other elements. The liquidus and solidus temperatures for various melting scenarios, involving combinations of elements, were calculated using the FactSage software, version 7.2 with the FTlite and FactPS databases to determine the optimal steps of the sequential process. All melting experiments were conducted using ceramic crucibles of high purity alumina (Al_2_O_3_).

The chemical compositions of synthesized alloys were measured by Inductively Coupled Plasma Atomic Emission Spectroscopy: CAP-017U (ICP-AES). The internal integrity and microstructural homogeneity of ingots were examined using optical metallography. To map and quantify the elemental composition of alloys at a microscale a Scanning Electron Microscope (SEM) with Energy Dispersive X-Ray Spectroscopy were used. The computational thermodynamic analysis of phase composition under non-equilibrium (Scheil) cooling conditions was conducted using the FactSage software.

### 5.1. Mg-Rich None-Equimolar Mg_50_(MnAlZnCu)_50_ Alloy

Magnesium containing high-entropy alloys with five elements Mg_x_(MnAlZnCu)_100-x_ (x: atomic percentage; x = 20, 33, 43, 45.6 and 50) was designed with a Mg content of up to 50 at% [27]. The composition of the Mg_50_(MnAlZnCu)_50_ alloy that was selected for this study includes a density of 2.2 g/cm^3^, which is less than that of its equiatomic MgMnAlZnCu counterpart [28].

#### 5.1.1. Synthesis Procedure

According to FactSage analysis, two-step synthesis requires melting, during Step 1, an intermediate Cu+Al+Mn alloy, i.e., without Zn and Mg. Then, in Step 2 of the synthesis, the Step 1 alloy is combined with Zn and Mg, thus reaching the final alloy chemistry:

Step 1: Cu + Al + Mn liquidus 972 °C (measured).
Step 2: Step 1 + Zn + Mg liquidus 1088.35 °C (FactSage-estimated).

The liquidus of the Step 2 alloy is just above the predicted boiling point of Zn and just below the predicted boiling point of Mg, which at 1 atm are located at 907 °C and at 1091 °C, respectively. Hence, the loss of Zn due to evaporation would change the alloy composition enough for the solid phase to re-precipitate.

The Step 1 alloy was synthesized under atmospheric pressure with inert gas argon blanketing. The ingredients consisted of Al, being a good metallic solvent, along with Cu and Mn, high melting point elements with low vapor pressure. The element recovery of 100% was used for Al and Mn while 95% was used for Cu. During Step 1 synthesis, the alloy with the target composition of Al_18.6_Cu_43.7_Mn_37.8_ (wt%) was aimed for, reaching the actual chemistry of Al_19.95_Cu_45.80_Mn_34.21_ (wt%) (Table 2). The low liquidus temperature of the Step 1 alloy positively affects the temperature and pressure requirements for Step 2 alloy synthesis.

During Step 2 melting, the appropriate amount of Step 1 alloy was combined with the two remaining elements of the target alloy, i.e., Mg and Zn. The target process temperature was 1100 °C, and argon pressure in the chamber was held at 0.55 MPa (80 psi). Recovery of the Step 1 alloy was estimated to be 100% while recovery rates for Mg and Zn were both estimated at 90% and therefore extra material was added to compensate for evaporation losses. After the molten Step 1 alloy reached the target temperature, Mg was added first due to its lower vapor pressure, followed by Zn. During the total alloying process that lasted 15 min, fourteen pieces were individually introduced to the melt through the airlock, allowing the melt temperature to recover after each addition. Due to low visibility inside the chamber, temperature data were instead used to verify when each piece reached the melt. During the experiment, argon was purged through the chamber at a low flow rate while maintaining the operating pressure. After the temperature recovered and an additional 30 s of stirring, the power was turned off, allowing the alloy to solidify. The sample weight control revealed that for the 137.5 g of the alloy retrieved, 9.3 g was lost which was more than the 8.1 g initially estimated.

The final composition of the synthesized alloy is listed in Table 2. It is seen that the contents of elements with the highest vapor pressure, Mg and Zn, exceed the target by 0.7 wt% and 1.53 wt%, respectively. The content of Mn exceeds the target by 3.56 wt%. At the same time, the content of Cu, the element with the lowest vapor pressure, is below the target by 4.83 wt%. The final chemical composition indicates that the Step 1 elements (Al, Mn, Cu) were low, while the Step 2 elements (Mg, Zn) were high. The higher-than-expected recovery rates of volatile elements points towards the high effectiveness of the PAIM process in preventing volatile element loss. The results of this synthesis can be used to further adjust element recovery rates for more precision in subsequent experiments.

#### 5.1.2. Alloy Characteristics

**Figure 6:**
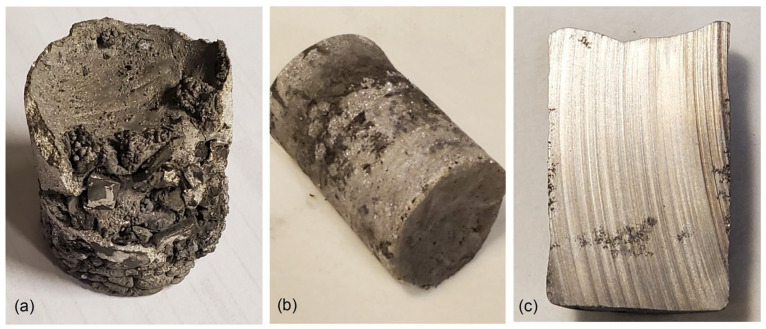
Ingots of the Mg_50_(MnAlZnCu)_50_ alloy synthesized using the PAIM furnace: (**a**) an initial trial ingot with heavily burnt and oxidized surface; (**b**) ingot with improved synthesis parameters; (**c**) internal integrity of an ingot seen on the cross-section. Ingot diameter—34 mm.

**Figure 7:**
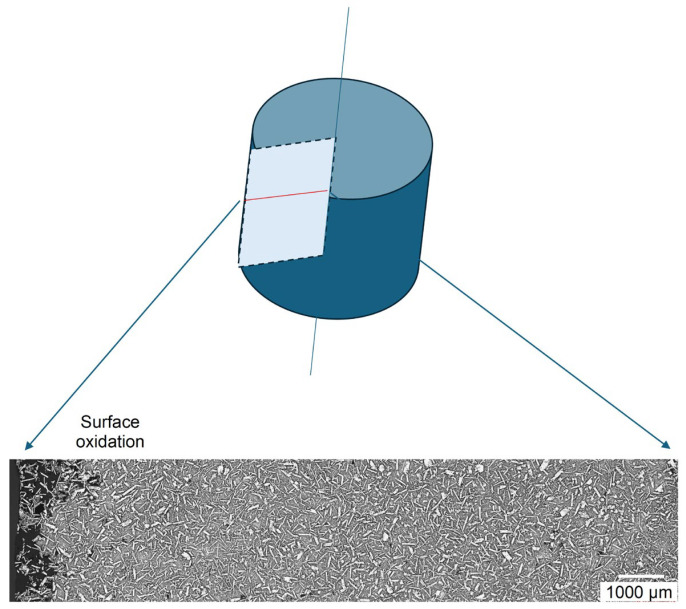
Microstructure of the Mg_50_(MnAlZnCu)_50_ alloy revealed on the ingot cross-section indicating high homogeneity. Some oxidation of the surface layer is present.

The Mg_50_(MnAlZnCu)_50_ alloy, due to its high Mg content, is highly reactive with oxygen. This is shown in Figure 6a, when during the initial trial of synthesis insufficient protection by inert gas argon was provided and the ingot experienced oxidation and burning. When argon protection was improved, surface oxidation was substantially reduced (Figure 6b). As seen on the ingot cross-section, there is no localized shrinkage or evident microporosity, randomly distributed on the ingot cross-section (Figure 6c). The ingot cross-section was also subjected to microscopic examinations, which revealed that the alloy is effectively fully dense and chemically homogenous (Figure 7). No compositional gradient is seen across the ingot cross-section. Some surface degradation occurred due to the solid-state reaction with oxygen.

**Figure 8:**
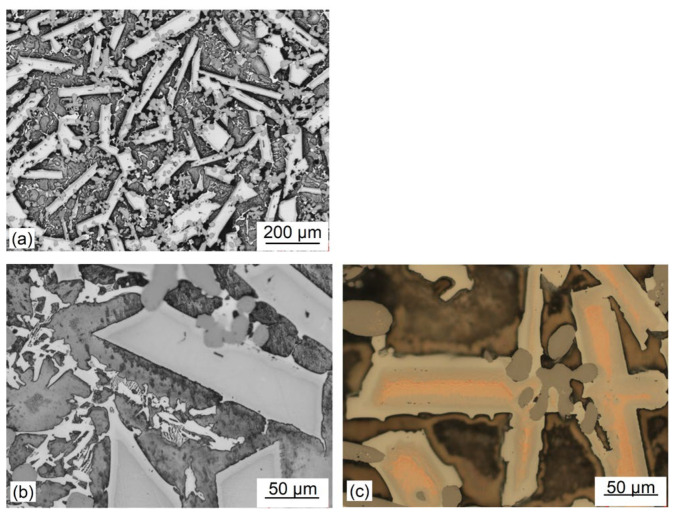
Microstructure of the Mg_50_(MnAlZnCu)_50_ alloy: (**a**) low magnification showing microstructure homogeneity along the ingot cross-section; (**b**) microstructure details; (**c**) color imaging with a contrast revealing the segregation gradient within the coarse laths.

The alloy microstructure consists of a matrix with a coarse lath-shape phase with bright contrast and a length of about 500 µm. It appears that the laths are oriented randomly (Figure 8a). The higher magnification revealed internal precipitates of different sizes and shapes, mainly within matrix (Figure 8b). As revealed by polarized light imaging, the laths exhibit a layered structure suggesting the chemical composition gradient (Figure 8c).

**Figure 9:**
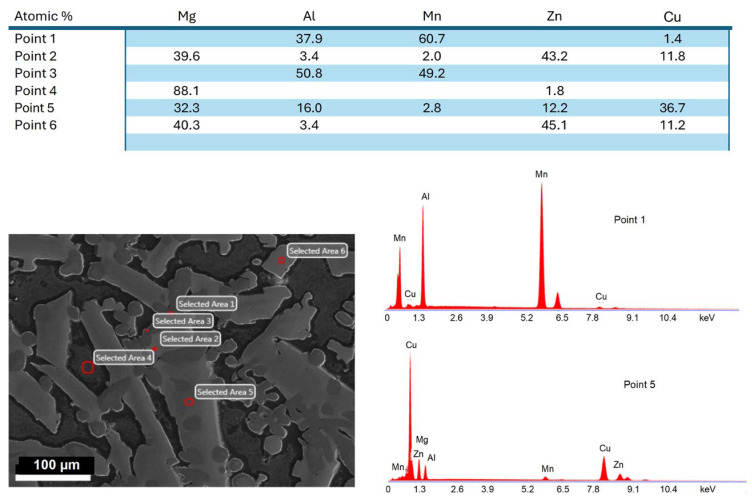
SEM-EDX point analysis of the Mg_50_(MnAlZnCu)_50_ alloy: microstructure image with locations of analyzed regions and table listing EDX results of elemental compositions of phases indicated in the image. Examples of EDX spectra with locations indicated.

**Figure 10:**
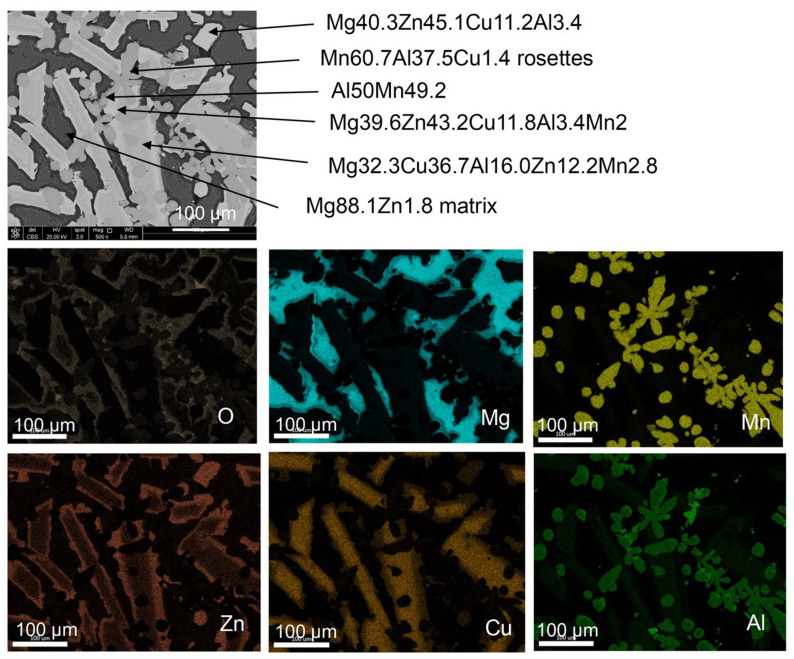
SEM image with phases deduced and EDX analysis of the Mg_50_(MnAlZnCu)_50_ alloy showing surface elemental distribution of alloying elements (at%).

The EDX elemental analysis using SEM allowed us to determine the approximate chemical compositions of the major phases distinguished based on morphology differences. The approximate compositions along with corresponding locations are listed in Figure 9. To reveal the phase distribution within the alloy the EDX point analysis was combined with EDX area mapping of the distribution of alloying elements. The summary is shown in Figure 10. The alloy matrix is Mg with traces of Zn and, as indicated by the oxygen distribution detected by EDX, regions are covered by a thin oxide film. The lath-shape phase consists of Cu and Zn-rich phases with a perimeter depleted in Cu, i.e., enriched in Zn. The randomly distributed rosettes contain Al and Mn. This preliminary phase analysis based on EDX chemical composition shows differences between our measurements and the FactSage software prediction for Scheil cooling conditions with non-equilibrium solidification (Table 3).

### 5.2. Al-Rich None-Equimolar Al_35_Mg_30_Si_13_Zn_10_Y_7_Ca_5_ Alloy

The aluminum-rich high-entropy alloy with six elements Al_35_Mg_30_Si_13_Zn_10_Y_7_Ca_5_ has a density of 2.73 g/cm^3^ and was originally synthesized by disintegrated melt deposition (DMD) with no further experimental details provided [29,30]. Although Al is prevalent, the alloy contains high fractions of Mg and Zn.

#### 5.2.1. Synthesis Procedure

According to the FactSage prediction of melting and boiling temperatures, the alloy melting is divided into 2 steps with liquidus temperatures specified below:

Step 1: Al + Si liquidus: <900 °C.
Step 2: Step 1 + Zn + Y + Ca + Mg liquidus: 1156.19 °C (FactSage).

As predicted by FactSage, during Step 2 melting, the formation of the high temperature solid phase Y_3_Si_5_ increases the Step 2 alloy liquidus temperature well above the boiling points of Zn (907 °C) and Mg (1091 °C) potentially causing their large losses.

The experimental synthesis of the Al-rich alloy was conducted by using all assumptions and recoveries determined during the melting of the Mg-rich alloy. The recovery rates were less than 100% for Mg and Zn due to vapor losses and for Y and Ca due to expected oxidation losses, despite the inert atmosphere. The overall procedure followed the Mg-rich alloy synthesis very closely, as described in Section 5.1.1. Step 1 involved melting Al, as a good metallic solvent, and a non-volatile, high-melting-point element, Si, with argon blanketing under atmospheric pressure. An attempt to also include Y and Ca, elements highly reactive with oxygen but not volatile, in Step 1 was unsuccessful. To correct the first attempt, Y and Ca additions were moved to Step 2, and the whole process was repeated. The element recovery rates were set as 100% for Al and Si, 97% for Y and Ca, and 95% for Mg and Zn. The two latter ones were determined during Mg-rich alloy synthesis.

During Step 2 melting, the Step 1 alloy was placed in the PAIM furnace crucible along with Y and the melting sequence started with the chamber being vacuum prepared, then pressurized with argon at 0.83 MPa (120 psi). Once the content was melted and the Y dissolved, around 1510 °C, the temperature was reduced to 1330 °C for Ca and Mg addition. Following that, the melt temperature was further reduced to 1250 °C and the chamber argon pressure was raised to 1.03 MPa (150 psi). After the addition of Zn, its melting and brief holding, the power was turned off to allow the alloy to solidify. During solidification the chamber pressure was maintained. The additions of Ca, Mg and Zn took 9 min. The overall Step 2 process lasted approximately 1 h, mainly to complete the melting of Y at a high temperature above 1500 °C. During the synthesis of Al-rich LWHEA, 522 g of source material was used to generate 500 g of alloy. The post-solidification weight control revealed that the final sample reached only 445 g, indicating that all the elements’ losses have not been fully compensated for during extra material addition.

The final composition of the synthesized alloy is shown in Table 4. The major outcome is a larger deviation from the target than that observed for Mg-rich alloy. It means that recoveries determined for Mg-based alloy did not allow us to reach the target chemistry of the Al-rich alloy. All synthesis parameters, including the process time, temperature, and pressure, since they all influence the alloy recovery, will need to be adjusted for better synthesis precision. The results of this synthesis can be used to further adjust the element recovery rates for more precision in subsequent experiments.

#### 5.2.2. Alloy Characteristics

**Figure 11:**
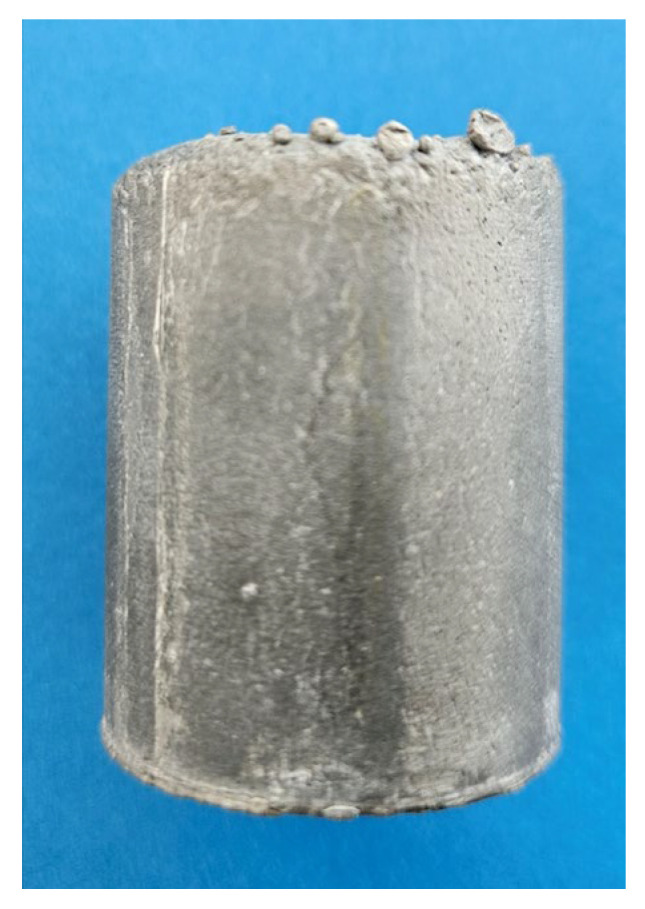
As-solidified ingot of the Al_35_Mg_30_Si_13_Zn_10_Y_7_Ca_5_ alloy synthesized using the PAIM furnace. Ingot diameter—34 mm.

The Al_35_Mg_30_Si_13_Zn_10_Y_7_Ca_5_ alloy containing Mg is less prone to surface oxidation than the Mg-rich alloy; this is supported by the bright shiny surface of the ingot after solidification (Figure 11).

**Figure 12:**
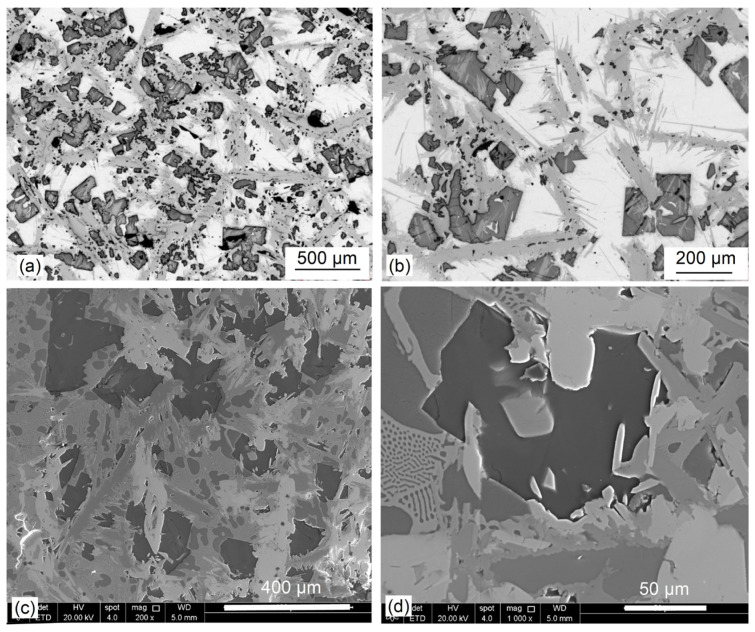
Microstructure of the Al_35_Mg_30_Si_13_Zn_10_Y_7_Ca_5_ alloy: optical images of general view (**a**) and morphology of alloy phases (**b**); SEM images showing general view (**c**) and detailed phase morphology (**d**).

The alloy microstructure when observed at low magnifications exhibits three different morphologies: a dark contrast blocky phase, gray contrast coarse laths with a length of 500–700 µm and width of about 50 µm, and a white contrast matrix (Figure 12a,c). At higher magnification of the optical microscope, the matrix and laths are not seen to be homogeneous but contain fine precipitates of irregular shapes (Figure 12b). The substructure details are clearly seen under SEM contrast, where regions of the matrix are covered by eutectic morphologies (Figure 12d).

**Figure 13:**
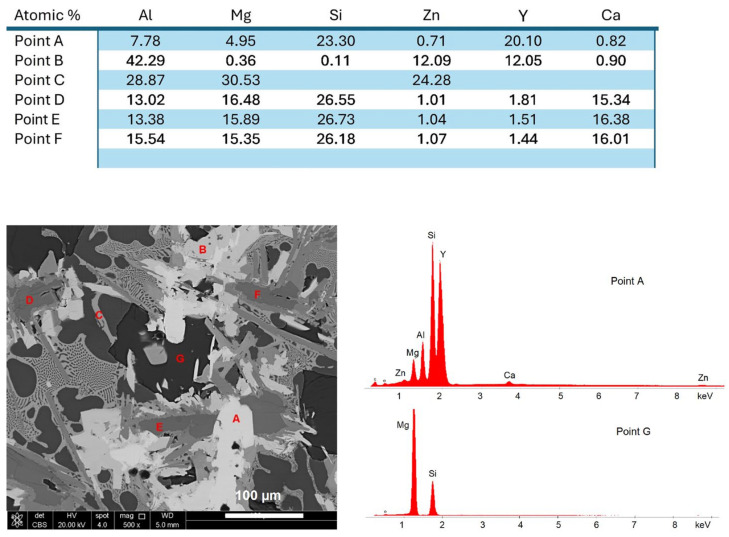
SEM-EDX point analysis of the Al_35_Mg_30_Si_13_Zn_10_Y_7_Ca_5_ alloy: microstructure image with locations of analyzed regions and table listing EDX results of elemental compositions of phases indicated in the image. Examples of EDX spectra with locations indicated.

The EDX elemental analysis revealed four major compositions indicated in the SEM image and listed in Figure 13. The dark blocky compounds correspond to the Mg_2_Si phase.

**Figure 14:**
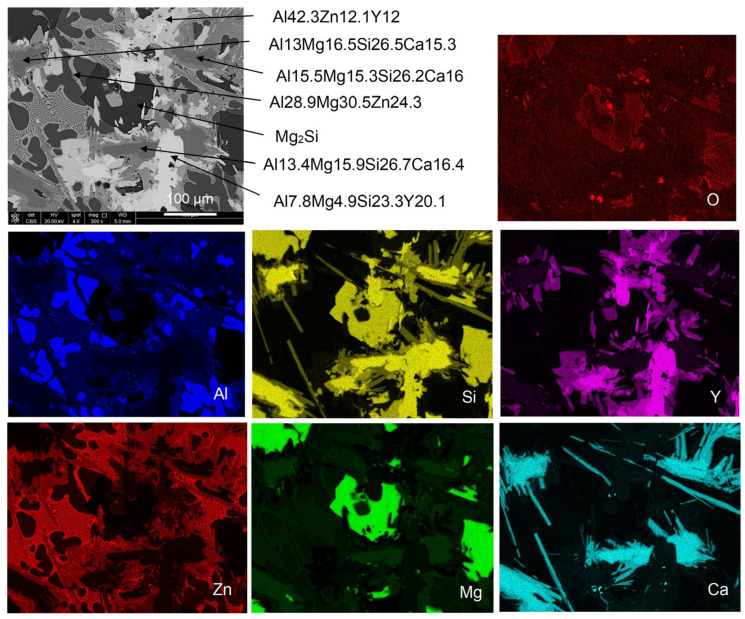
SEM-EDX area analysis of the Al_35_Mg_30_Si_13_Zn_10_Y_7_Ca_5_ alloy showing surface elemental distribution (at%).

The summary of EDX point analysis and area mapping of elemental distribution is shown in Figure 14. Similarly, as was the case for the Mg-rich alloy, some differences exist between the results of the EDX analysis and the FactSage software prediction for Scheil cooling conditions with non-equilibrium solidification (Table 5). Details of the phase composition that require crystallographic analysis will be published separately.

## 6. Assessment of the PAIM Equipment Performance and Synthesis Outcome

The rationale behind pursuing the project of new equipment design and built in this study was the lack of a commercially available system that could be used either directly or after modifications for the synthesis of LWHEA compositions. In addition to the gas pressure level and its control capabilities, a certain furnace size with an optimal volume for alloy discovery research was required. On the one hand, the synthesized alloy volume should resemble an ingot, showing features on mini-scale that are typical for its larger counterparts, e.g., solidification, shrinkage and cracking as well as segregation tendencies, and allow conclusions on the scalability potentials of the alloy synthesis. Moreover, it should have the volume sufficient for multiple laboratory tests of various properties, e.g., mechanical tests. On the other hand, the melt should consume the minimal volume of alloy ingredients. Such an approach optimizes the cost of alloy development projects that for the exploratory stage of HEAs often require testing a large number of chemical compositions. The number of tests conducted using the PAIM furnace positively proved the optimal volume determination.

The chemical composition nature of HEAs, where all elements are in near-equiatomic concentrations, requires different strategies during their synthesis through liquid routes than those commonly used for conventional alloys. In conventional alloys, the base metal content does not require special attention, e.g., the content of Fe in steel, since during their synthesis the goal is to achieve each alloying element content within its specified range, and the base element will appear as the “remainder”. In contrast, there is no base element in HEAs, and the concentration of each constituent element is equally important and requires individual attention. Therefore, if one element is too low, another one will automatically be too high. This issue also causes difficulties during the chemical analysis of HEAs since typical measuring instruments are also calibrated to measure the alloy chemistry under the assumption of a “remainder” base metal.

The deliberately selected complex mixtures of elemental constituents within the Mg_50_(MnAlZnCu)_50_ and Al_35_Mg_30_Si_13_Zn_10_Y_7_Ca_5_ alloys synthesized in this study were the key challenges to overcome. The alloy selection objective was to involve the latest global trends of LWHEA development. Although early HEAs relied on the theoretical capabilities of solid solutions with high thermodynamic stability, later compositions included rather complex systems with many phases, and this evolving trend is explored in modern LWHEAs where more complex average compositions are pursued. For both alloys, Mg and Zn are combined with low vapor pressure elements, Mn, Cu, Si, Y or Ca. The challenge is common since Mg is regularly added to LWHEAs to significantly reduce density and enhance their strength-to-weight ratio [4,6,22]. The magnesium boiling point of 1091 °C is well below the melting point of other constituents. Moreover, Mg exerts very high affinity for oxygen and to prevent oxidation an inert gas shield is required. Although Zn also creates challenges during synthesis, adding Zn improves castability and structural integrity thus helping the synthesis [31]. In Al-Zn-Mg-Cu HEA systems, Zn refines the microstructure and improves strength through solid solution strengthening and the formation of nano-precipitates (MgZn_2_) [32]. The experiment of this study showed that primarily the high Mg content rather than Zn contributes to the difficulties with alloy synthesis. Therefore, of two alloys of this study, the Mg-rich composition was more difficult to synthesize.

It is of interest that although the Mg_50_(MnAlZnCu)_50_ alloy was more difficult to synthesize, the experimentally determined element recovery rates allowed it to synthesize its elemental composition very close to the nominal target, with Mg and Zn contents exceeding the target. The higher-than-expected recovery rates of volatile elements points towards the high effectiveness of the PAIM process in preventing volatile element loss. On the other hand, the target composition deviation obtained during synthesis of the Al_35_Mg_30_Si_13_Zn_10_Y_7_Ca_5_ alloy, with lower than anticipated contents of Mg and Zn, emphasizes the need for reliable recovery rates of volatile alloy constituents. It shows that such recovery rates cannot be adopted from the synthesis of other alloy chemistries; instead, they must be experimentally determined for the specific alloy composition and synthesis parameters, including the processing (melting) time, temperature, and furnace gas pressure.

The detailed synthesis procedures of LWHEAs described in this report with fundamentals of liquid metal behavior should help fill the literature information gap on this subject and help materials scientists with their attempts at synthesizing complex and challenging LWHEA compositions. This is of particular importance for very popular discovery research programs of HEAs, where novel alloys are designed in large numbers through computer-aided methods and theoretical considerations, thus requiring experimental verification through laboratory alloy synthesis and examination of their structure at different levels.

## 7. Conclusions

To overcome the synthesis challenge of lightweight high-entropy alloys, consisting of elements with drastically different melting temperatures and vapor pressures, a novel pressure-assisted induction melting process was developed and the proprietary furnace for its implementation was designed and built. The system allows precision melting of up to 10 cm^3^ of an alloy at temperatures up to 1700 °C while addressing the partial pressure requirements during the melting progress, starting from a rough vacuum of about 10^−4^ MPa and balancing it by inert gas argon, introduced into the chamber up to a maximum pressure of 1.6 MPa (233 psi). The alloy chemical composition can be modified in situ at specific melting stages by feeding solid additives into the melt through the isolated airlock without disrupting the pressure conditions within the chamber. The size of a furnace pressure chamber with a total volume of 37 L (0.037 m^3^) allows for a further expansion of the synthesized alloy volume to 100 cm^3^.

The viability of the concept was verified by the synthesis of two lightweight high-entropy alloys: Mg-rich Mg_50_(MnAlZnCu)_50_ and Al-rich Al_35_Mg_30_Si_13_Zn_10_Y_7_Ca_5_. The experiments showed that the sequential multi-step synthesis process, designed based on inputs from FactSage computational analysis, when combined with PAIM synthesis, allowed the manufacturing of fully dense and chemically homogenous complex alloy compositions with optimal volumes for materials discovery research. Although the furnace gas pressure is essential in controlling the volatile element loss, for the precise reaching of the alloy nominal target, the recovery rates of alloying constituents must be determined experimentally for specific alloy compositions and PAIM synthesis parameters.

## Figures and Tables

**Table 1:** Characteristics of the elements in LWHEAs synthesized in this study with several others added as a reference [21,22,23].

Element	Atomic Mass	Melting Point	Boiling Point	Enthalpy of Vaporization	Density
		°C	°C	kJ/mol	g/cm^3^
Li	6.941	180	1342	136	0.53
Mg	24.305	650	1091	132	1.74
Al	26.981	660	2470	284	2.7
Si	28.085	1414	3265	383	2.33
Ca	40.078	842	1484	155	1.53
Ti	47.880	1668	3287	427	4.51
Cr	51.996	1907	2672	347	7.19
Mn	54.938	1246	2061	225	7.21
Fe	55.847	1538	2861	354	7.87
Cu	63.546	1084	2562	305	8.96
Zn	65.390	419	907	115	7.14
Y	88.905	1526	3345	363	4.47
Ce	140.116	795	3443	398	6.77

**Table 2:** Nominal and measured compositions of the Mg_50_(MnAlZnCu)_50_ alloy (Method: CAP-017U (ICP-AES).

		Mg	Mn	Al	Zn	Cu	Remarks
Nominal (target)	at%	50	12.5	12.5	12.5	12.5	
Nominal (target)	wt%	31.56	17.83	8.76	21.22	20.63	
1 Step alloy measured	wt%	0.01	34.21	19.95	0.01	45.80	Si 0.02; Pb,Sn 0.01; P,Fe,Ni,Sb,Co < 0.01
2 Step alloy (final) measured	wt%	32.26	21.39	7.75	22.75	15.80	Fe 0.05; P 0.01; Si,Pb.Sn,Sb, Co < 0.01

**Table 3:** Major phases in the Mg_50_(MnAlZnCu)_50_ alloy predicted by the FactSage software and identified in the alloy synthesized.

Atomic %	FactSage Phase Name	Crystal Structure	Predominant Chemistry (at%)	Phase Present in Synthesized Alloy (at%)
50.266	HCP	A3	Mg, 17%Mn, 11%Al	(4) Mg_88.1_Zn_1.8_ matrix
39.702	Laves C15	C15	Cu_2_Mg, Zn_2_Mg, Al_2_Mg	(2) Mg_39.6_Zn_43.2_Cu_11.8_Al_3.4_Mn_2_
				(5) Mg_32.3_Cu_36.7_Al_16.0_Zn_12.2_Mn_2.8_
				(6) Mg_40.3_Zn_45.1_Cu_11.2_Al_3.4_
2.026	Mg_12_Zn_13_		Mg_12_Zn_13_	
2.101	CUB	A13	62%Mn, 38%Al	(3) Al_50_Mn_49.2_
0.941	BCC	A1	56%Mn, 41%Al, 2%Cu	(1) Mn_60.7_Al_37.5_Cu_1.4_ rosettes

**Table 4:** Nominal and measured compositions of the Al_35_Mg_30_Si_13_Zn_10_Y_7_Ca_5_ alloy (Method: CAP-017U (ICP-AES).

		Al	Mg	Si	Zn	Y	Ca	
Nominal (target)	at%	35	30	13	10	7	5	
Nominal (target)	wt%	26.87	20.74	10.39	18.60	17.70	5.70	
2 Step alloy (final) measured	wt%	25.58	14.57	8.04	16.93	19.55	5.37	
2 Step alloy (final) impurities	wt%	Fe 0.04 Cu 0.03; Ag, As, B, Ba, Be, Bi, Cd, Ce, Co, Cr, In, K, Li, Mn, Mo, Na, Nb, Ni, P, Pb, Pd, Pt, Sb, Se, Sn, Sr, Ta, Te, Ti, Tl, V, W and Zr < 0.01%						

**Table 5:** Major phases in the Al_35_Mg_30_Si_13_Zn_10_Y_7_Ca_5_ alloy predicted by the FactSage software and identified in the alloy synthesized.

Atomic %	FactSage Phase Name	Crystal Structure	Predominant Chemistry	Phase Present in Synthesized Alloy (at%)
36.552	Prototype_Mg_32_(Al,Zn)_49_	D8e	Mg_32_(Al,Zn)_49_	(C) Al_28.9_Mg_30.5_Zn_24.3_
21.663	Prototype_CaF_2_	C1 (anti-fluorite)	Mg_2_Si with trace Al	(G) Mg_2_Si
12.740	Prototype_Al_4_Ba	D13	Al_4_Ca, Zn_4_Ca with trace Mg, Si	—
11.617	Prototype_CrB	B33	YSi with trace Al, Ca	(A) Al_7.8_Mg_4.9_Si_23.3_Y_20.1_
6.404	Laves_C15	C15	Al_2_Ca, Al_2_Y, Al_2_Mg, with trace other elements	
3.799	Al_3_Y_Ni_3_Sn	D019	Al_3_Y pure phase	

## Data Availability

The original contributions presented in this study are included in the article. Further inquiries can be directed to the corresponding author.
